# The Influence of Finishing on the Pilling Resistance of Linen/Silk Woven Fabrics

**DOI:** 10.3390/ma14226787

**Published:** 2021-11-10

**Authors:** Eglė Kumpikaitė, Indrė Tautkutė-Stankuvienė, Lukas Simanavičius, Stasė Petraitienė

**Affiliations:** 1 Department of Production Engineering, Faculty of Mechanical Engineering and Design, Kaunas University of Technology, Studentų str. 56, LT-51424 Kaunas, Lithuania; indre.stankuviene@gmail.com (I.T.-S.); lukas@klt.lt (L.S.); 2 Department of Applied Mathematics, Faculty of Mathematics and Natural Sciences, Kaunas University of Technology, Studentų str. 50, LT-51424 Kaunas, Lithuania; stase.petraitiene@ktu.lt

**Keywords:** linen/silk fabric, pilling resistance, singeing, pigment and reactive printing

## Abstract

The pilling resistance of fashion fabrics is a fundamentally important and frequently occurring problem during cloth wearing. The aim of this investigation was to evaluate the pilling performance of linen/silk woven fabrics with different mechanical and chemical finishing, establishing the influence of the raw material and the peculiarities of dyeing and digital printing with different dyestuff. The pilling results of the dyed fabrics were better than those of the grey fabrics and even a small amount of synthetic fiber worsened the pilling performance of the fabric. Singeing influenced the change in the pilling resistance of the linen/silk fabrics without changing the final pilling resistance result. Singeing had a stronger influence on the fabrics with a small amount of synthetic fibers. The pilling resistance of printed fabrics was better than that of grey and dyed fabrics without and with singeing. The pilling resistance of pigment-printed fabrics was better than that of the reactive-printed fabrics.

## 1. Introduction

It is known within the practice of textile manufacturing that the finishing of woven fabrics, such as abrasion and pilling resistance, has a particularly strong influence on their end-use properties. Thus, it is highly relevant to solve the issue of decreasing the pilling resistance of woven fabrics before their use in everyday wearing.

Fabric pilling is considered to be a performance and aesthetic property of a woven product that determines its quality [1].

Pilling is a fabric surface defect that develops due to fiber movement or the slippage of yarns caused by abrasion and wear. Pilling occurs in four steps: fuzz formation, entanglement, growth, and wear-off. The formation of fuzz and pills influences the aesthetics and durability of the fabric, as well as the demand of consumers [2]. Abrasion and pilling resistance can be established using the Martindale abrasion tester, which generates a movement according to the Lissajous curve and can test several samples simultaneously [3].

The abrasion and pilling resistance of both woven and knitted fabrics have been investigated by a number of scientists. The properties of these fabrics are influenced by such factors as the raw material (the composition of the fibers, the amount of synthetic fiber in the yarn, etc.), the structure (woven and knitted fabrics, different weaves, yarn structure parameters, etc.), and the finishing (mechanical and chemical finishing, different coatings, etc.).

Different scientists have analyzed the influence of the raw material on abrasion and pilling resistance. It was established that a larger amount of polyester fiber in a cotton/PES blend reduces the pilling resistance of knitted fabrics [4]. The addition of PES, PA fibers, or elastane filaments to the structure of socks enhanced the abrasion resistance of the garment [5,6]. The percentage of the mass loss was higher for knitted samples from wool than that for cotton samples [7]. The pilling resistance of polyester/wool woven fabrics has also been investigated. The number of polyester fibers migrated on the surface of a yarn increased with an increase in the polyester content of the blend and, hence, the pilling increased. The converse was true for the wool fibers used in the blend [8,9]. Fabrics comprising 100% cotton had better pilling performance than blended cotton/PES fabrics but their abrasion resistance was the lowest [10]. In summary, it can be stated that the content of synthetic fibers has a significant influence on the abrasion resistance and pilling performance of fabrics. Blends of natural and synthetic fibers have been investigated widely, whereas blends of two natural fibers of a different nature—linen (cellulose fiber) and natural silk (protein fiber), for example—have not been studied. This is a new blend of two natural fibers in one yarn and it has not been studied in earlier research.

The pilling and abrasion resistance also depend on the yarn structure. The length and fineness of the component fibers, as well as the yarn twist, density, and type of the weave of the fabric, influence pilling resistance [11,12]. The use of yarns with a higher linear density increased the abrasion resistance of knitted socks [5], polyester/wool [8], and polyester/cotton [13] woven fabrics. When the twist of the fabrics is higher, the relative slippage between the fibers decreases and the hairiness floating on the surface of the fabric is less. Therefore, the tendency to pill gradually decreases [12]. The abrasion and pilling resistance of fabrics produced from compact yarns were higher than those of fabrics produced from ring yarns [14], and they were lower in fabrics from carded yarn than in fabrics from combed yarn [15]. Pilling grades, from low to high, are ring-spun fabric, siro-spun fabric, compact-spun fabric, and siro–compact-spun fabric, respectively [12]. Yarn structure is also an important parameter when pilling performance is studied. In this research, the fiber composition and structure of the fabric of the yarn were constant.

The structure of the fabric is another important factor involved in pilling and abrasion resistance. Knitted fabrics of a different structure have been frequently analyzed by scientists. The abrasion resistance and pilling performance of interlock fabrics were higher than those of jersey fabrics [14]. Single jersey knitted fabrics showed improved resistance compared to rib and moss stitch structures [7]. Abrasion resistance depends on the structure of knitted fabric parameters, such as the stitch length: the abrasion resistance decreases when the stitch length increases [15,16]. Woven fabrics exhibited better pilling performance than knitted ones [7]. The pilling performance of a woven fabric depends on the float length, i.e., weaves with shorter floats have better pilling resistance than those with long floats [8,13,17]. Fabric woven at a proper loom setting or warp yarn tension showed higher strength, less pilling, and a superior abrasion tendency when compared with fabrics woven at various other levels of warp yarn tension [18]. The structure of the woven fabrics used was always the same—the raw material, structure, and linear density of the warp and weft, warp and weft settings, and weave—in all investigated fabrics.

The greatest amount of research has analyzed the influence of finishing on abrasion and pilling resistance. Processes such as singeing, cropping, and heat setting significantly reduce the tendency to pill [8,11]. Mechanically singed samples exhibited a better pilling grade than samples without singeing [4,8,9,12,19]. The abrasion resistance and pilling performance of dyed fabrics were higher than those of loom state fabrics [6,14,19]. Pilling resistance significantly increased with the wool content in polyester/wool fabrics and the heat setting temperature [20]. The tendency to pill of a CVC knitted fabric could be reduced by singeing and the heat setting [21]. Different coatings also affect the improvement of abrasion resistance and pilling performance [22,23,24]. A crease-resistant finishing applied to apparel fabric improved the usage characteristics of clothes and enhanced the pilling performance of woven fabrics [25]. Different mechanical and chemical means were used for the woven fabrics investigated in the article. One of these finishing methods was new and another had already been used by other researchers but, on the whole, the focus was on investigating the pilling performance of fabrics. Loom state, dyed, singed, and printed woven fabrics are investigated in this paper. The pilling performance of printed fabrics has not been investigated in any earlier scientific work; thus, the behavior of the fabric during the pilling test is important, especially the comparison of active and pigment printing. These two methods differ by their nature and technology, so it is relevant to establish and compare their pilling performance.

One more novelty of this investigation is the use of a mathematical analysis to establish the start of the pilling process, a new approach in textile research.

To deal with the information gap, the aim of the present article is to investigate the pilling performance of linen/silk woven fabrics with different mechanical and chemical finishing, establishing the influence of the raw material and the peculiarities of dyeing and digital printing with different dyestuff.

## 2. Experiment

### 2.1. Object of the Investigation

**Figure 1:**
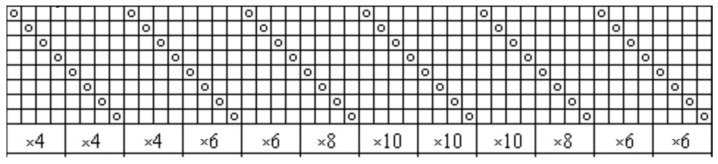
Drawing-in scheme of the woven fabric.

**Figure 2:**
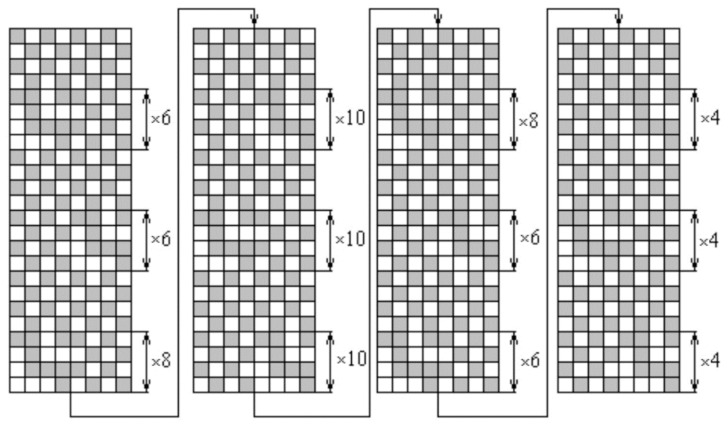
Cards of the woven fabric.

The object of the investigation was a woven fabric from a single blended yarn, 26 tex, 70% linen/30% silk in the warp and in the weft. The warp setting was 220 dm^−1^ and the weft setting was 223 dm^−1^. Another fabric used for comparison was woven from linen 28 tex yarn in warp and cotton/PES twisted 28 tex threads in weft. The fibrous composition of this fabric was 86% linen/12% cotton/2% PES. Both fabrics were woven using a double-layer weave with a one-layer weave. The warp and weft repeats of the weave were large and a general plan of the weave could cover a large area in the article. For this reason, the schemes of drawing-in and the cards of the fabric are shown separately. The weave was chosen because of the existence of one-layer (dense) and two-layer (rare) places in one fabric. Such a fabric structure allowed for the evaluation of the pilling performance in rarer and denser parts of the fabric. The fabric drawing-in scheme is shown in Figure 1 and the cards of the woven fabrics are presented in Figure 2. The fabrics were woven by the textile company Klasikinė tekstilė (Lithuania).

The fabrics were treated with different finishings. At first, grey fabrics and dyed fabrics without any additional finishing operations were investigated. After this, grey and dyed fabrics after singeing were analyzed. Two types of printed fabric (pigment and reactive printing) were also researched.

### 2.2. Finishing Materials and Technologies

All the finishing procedures (washing, dyeing, rinsing, softening, and drying) were performed in a BRONGO 100 (Brongo srl, Florence, Italy) machine. The fabrics were washed for 10–15 min at a temperature of 65 °C and dyed for 75–120 min at a temperature of 60 °C. Active dyestuff *Everzol* (Everlight Chemical, Taipei, Taiwan) was used. The fabrics were rinsed in cold water twice and in hot water twice after dyeing. One session of rinsing took 5 min. The softening was performed in an acid environment using a *Perustol CCF* (Rudolph Group, Gerestried, Germany) softener.

The singeing of the loom state fabrics was performed in a *Vollenweider* gas singeing machine (Xetma Vollenveider, Waedenswil, Switzerland) with an open flame. Two different sets of burners were used for singeing both sides of the fabric by threading the fabric suitably. In the gas singeing machine, the fabric in an open width passed at a speed of 55 m/min.

Both pigment and reactive digital printing were achieved with a piezoelectric DOD ink head. The piezoelectric material was placed in an ink-filled chamber behind each nozzle. When a demand (impulse) is applied, this special material changes shape, which generates a pressure pulse. This effect allows the ink fluid to exit the nozzle. This means that any type of ink can be used for printing with the same print head. Pigment printing was performed in a *Mimaki* printing machine (Mimaki Engineering Co., Ltd., Tomi, Japan) and reactive printing was conducted in an *Mtex500* printing machine (Techno Fashion World, Milano, Italy).

For the experiment, we used both pigment dyestuff and reactive dyestuff. The pigment and reactive dyestuffs were manufactured by *Mimaki* (Mimaki Engineering Co., Ltd., Tomi, Japan). The difference between the two inks is fundamental. Pigment dyestuff contains a binder in the dyestuff and thus the fabric does not need any special preparation before printing commences. Reactive dyestuff inks do not possess this feature. Fabrics need to be specially prepared for the print; they are soaked in a combination of chemicals that controls their color intensity, background color, and line sharpness. All the ingredients are fundamentally important but urea plays a key role in the soaking process. Urea is responsible for the color intensity and it must fulfill a high set of requirements for the drying process. The other major difference is the fixation process. Printed fabrics with pigments need a completely dry fixation and do not require washing afterwards; when using reactive dyestuff, a steaming process is required and washing afterwards is a necessary step.

### 2.3. Weather Conditions of the Experiment

The samples were laid on a plain horizontal surface. Air could pass through the fabric. The samples were conditioned for at least 24 h in standard weather conditions (Standard LST EN ISO 139: 2005/A1: 2011) before testing, i.e., the temperature was 20 ± 2 °C and the relative humidity was set at 65 ± 4%.

### 2.4. Methods of Establishing Pilling Resistance

**Figure 3:**
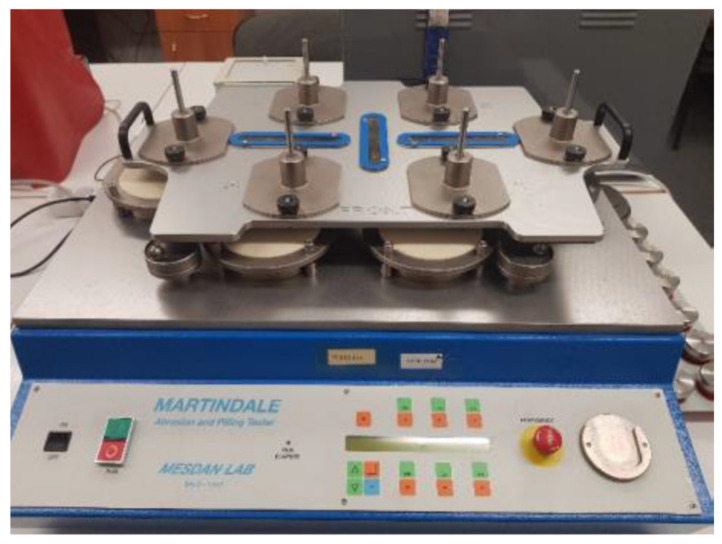
MESDAN-LAB, Code 2561E Martindale abrasion and pilling tester.

The pilling resistance tests were performed using a MESDAN-LAB, Code 2561E (SDL Atlas, Rock Hill, UK) Martindale abrasion and pilling tester according to Standard ISO 12945-2:2000 “Determination of fabric propensity to surface fuzzing and to pilling—Part 2: Modified Martindale method”. A picture of the tester is shown in Figure 3.

Six circular samples, from which three were placed on holders and another three were placed on the pilling table, were cut from the investigated fabrics. Each sample was evaluated by three experts according to an organoleptic evaluation after a certain number of cycles as specified in the standard (125, 500, 1000, and 2000 cycles). The marks of pilling of each sample were recorded and the average result of all the evaluations was established after the evaluation of the sample appearance. The evaluation of the pilling marks is described in Table 1.

### 2.5. Mathematical Analysis of the Results

A mathematical analysis was performed using MATLAB software.

## 3. Results and Discussion

**Figure 4:**
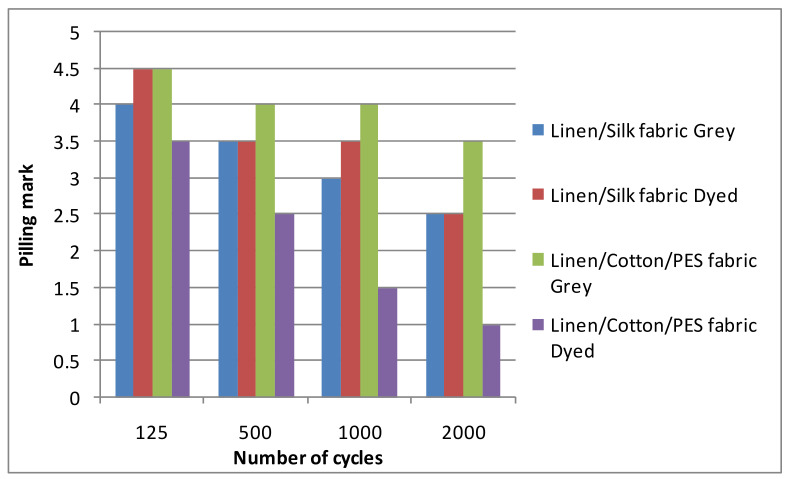
Pilling marks of grey and dyed linen/silk fabrics without additional mechanical finishing.

At first, grey and finished woven fabrics without any additional finishing were tested whilst seeking to establish the influence of the finishing on the fabric pilling resistance. The results are shown in Figure 4. The number of cycles was set during the experiment and the pilling and abrasion tester stopped after a set number of cycles. For this reason, a statistical analysis of the number of cycles cannot be provided. As can be seen, the results of the grey and dyed linen/silk fabrics were similar. Only at the beginning of the pilling test (125 cycles) was the mark of the dyed linen/silk fabric slightly (by 0.5) higher than that of the grey fabric. In the middle of the pilling test (after 500 and 1000 cycles), the marks were essentially the same, i.e., the score was 3.5 for linen/silk fabric. At the end of the test, the pilling mark was the same; it was equal to 2.5 for both the grey and dyed linen/silk fabrics. It can be seen from the diagram that the mark of the grey fabric changed gradually after each abrasion period. The character of the dyed fabric in the diagram was slightly different, i.e., after 125 abrasion cycles, the changes in the fabric surface were not significant. After 500 cycles, the mark decreased significantly (by 1 point) and during the next abrasion period (1000 cycles), it did not change again. At the end of the test (after 2000 cycles), a significant change (by 1 point) could be seen again. The pilling marks of the dyed linen/silk fabrics were higher than those of the grey fabrics. The results of the linen/cotton/PES fabric differed from the results of the linen/silk fabrics, i.e., the pilling performance of grey fabric was better than that of linen/silk fabric. However, the pilling marks of dyed linen/cotton/PES fabric were significantly lower than those of linen/silk fabric. Thus, it can be stated that even a small amount of synthetic fiber worsens the pilling performance of the fabric. The result of investigation [9] also showed the same tendency, i.e., the pilling performance of the dyed fabrics was higher than that of the loom state fabrics. Only investigations of cotton (cellulose fiber) [4,5,7,21,25] and wool (protein fiber) [6,23,24] and their blends with synthetics were found in the scientific literature. The tendencies of the results were similar because the raw material of the analyzed fabrics was of a similar nature (linen, cellulose fiber; natural silk, protein fiber). The reason for these results could be that the dyestuff seemed to adhere the formed fuzzes and pills to the surface of the fabric; the pilling resistance of the dyed fabric then improved. The used dyestuff may have had an influence on the pilling performance of the analyzed fabrics because reactive dyes form covalent bonds with the fabric. This situation was not analyzed in this article because it is not the object of textile engineering. A statistical analysis of the results cannot be provided because the result of the evaluation was marked; it could only be 1, 1.5, 2, 2.5, 3, 3.5, 4, 4.5, or 5, and no errors or other statistical parameters could be calculated for the pilling marks.

**Figure 5:**
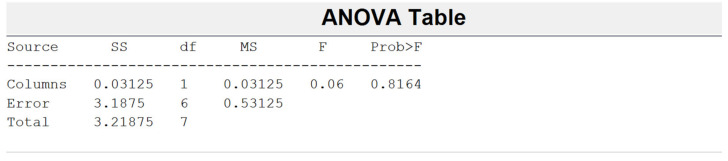
ANOVA results of singed fabrics.

When seeking to find a method for better pilling resistance, a type of additional mechanical finishing—singeing—was performed on the grey fabric. The singeing process improves the surface of fabric and its pilling resistance by removing protruding fibers from it [4,8,14]. The fabric was also dyed after singeing. The diagrams of the pilling resistance of the grey and finished linen/silk fabrics after singeing are shown in Figure 5. According to reference [9], the pilling performance of dyed linen/silk fabrics was higher than that of grey fabrics. Comparing the given results with the results of linen/cotton/PES fabrics, it can be seen that results of grey fabrics were better than those of linen/silk fabrics, but they remained the same as the results without singeing. The pilling performance was almost the same as the results of linen/silk fabrics, but they were much better than the results before singeing. Thus, it can be stated that singeing had a greater influence on the fabric with a small amount of synthetic fibers. The pilling resistance of different raw material fabrics after singeing differed. Most investigations are related to woven and knitted fabrics from cotton (cellulose fiber) [4,5,7,21,25] and wool (protein fiber) [6,23,24] and their blends with PES, PA, and elastane. It was established that the pilling performance of 100% natural fiber fabrics was higher than that of blends with synthetic fibers [4,5,6,7,21,23,24,25]. A blend of two natural fibers (linen and natural silk; cellulose and protein fibers) was analyzed in this article. The pilling performance influenced by the singeing process differed from the results of the blends with synthetic fibers. In summary, the results showed that singeing did not have a significant influence on the pilling resistance of blends of two natural fibers. This was also confirmed by the ANOVA statistical method using MATLAB software (Figure 5). If 0.8164 > 0.06, the hypothesis about the equality of averages was accepted and averages of groups did not differ statistically.

**Figure 6:**
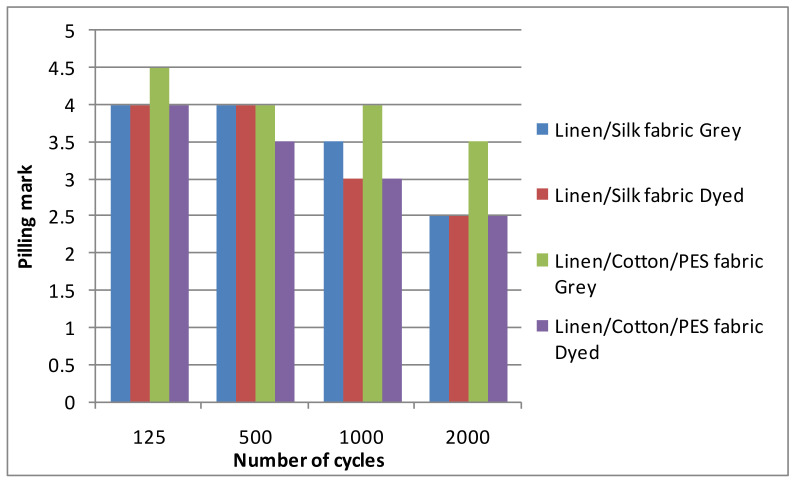
Pilling marks of grey and dyed linen/silk fabrics with additional mechanical finishing.

**Figure 7:**
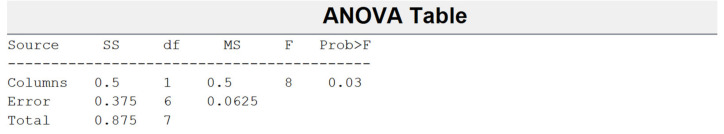
ANOVA analysis results for printed fabrics.

As can be seen from the diagram in Figure 6, the nature of the pilling diagrams for both the grey and finished fabrics was almost the same, i.e., almost all the marks were the same after a certain number of abrasion cycle periods. In comparison, the diagrams depicting the experiments without and with rinsing highlighted that the nature of the diagram was different. At first, the mark reached the level of mark 4 and the level did not change after 125 and 500 abrasion cycles. In the next two abrasion periods, the appearance of both the grey and dyed fabrics changed significantly. The final result of the pilling test of the fabrics after the singeing treatment was the same as that of the fabrics without singeing. It can be stated that the results of pilling resistance improved because the nature of the diagrams was better for the fabrics after singeing, i.e., those fabrics preserved a higher mark of pilling resistance for longer during the abrasion cycles. In addition, it could be seen that better results of pilling resistance were obtained for the grey fabric after singeing than for the dyed fabric. According to reference [8], these results are because the dyed fabric suffered a greater number of mechanical effects during the dyeing process, which worsened the general pilling resistance of the dyed fabric. The fabric weave can also have an influence on the pilling performance of the fabric. The two-layer (rare) parts form pills quicker than one-layer parts because the length of the floats is longer in two-layer parts. These results correspond with earlier investigations [9,23,24,25], which state that shorter thread floats result in the better pilling performance of the fabric. The fabric was woven from rotor-spun yarn and the length of the fiber was short. Due to this, shorter fibers fuzzed on the surface of the dyed fabric after the dyeing process and this influenced the pilling performance of the dyed fabric. Thus, singeing exerted an influence on the pilling resistance of the fabrics but it was not significant. In a comparison of the results of the fabrics without and with singeing, it could be seen that singeing improved the pilling performance of both the grey and dyed fabrics but the influence of the dying process was more significant because the results showed that the grey and dyed fabrics changed in nature. In conclusion, this type of mechanical finishing needs to be used as an additional finishing for better pilling resistance. Using the ANOVA analysis for the data from Figure 6, it was established that 0.03 > 8, and it could be concluded that the hypothesis about the equality of averages was canceled. This means that the results differed (Figure 7).

**Figure 8:**
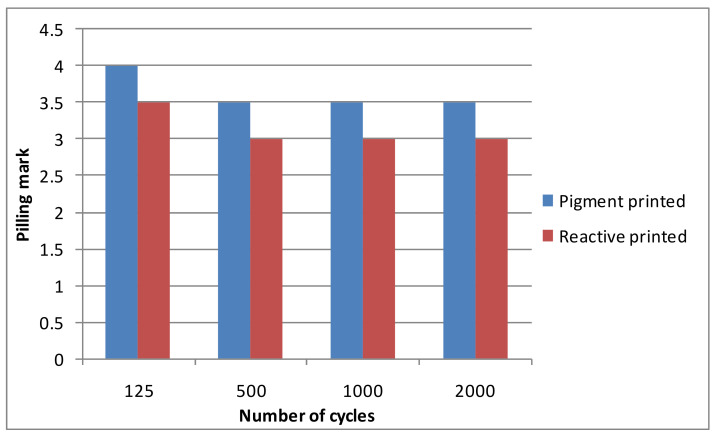
Pilling resistance of linen/silk fabrics after pigment and reactive printing.

Finishing such as digital printing has become popular in textile finishing. Thus, the pilling resistance of two types of digital printing—pigment printing and reactive printing—was analyzed in this study. The diagrams of the pilling resistance of both types of printing are presented in Figure 8.

As can be seen in Figure 6, the mark of the pilling resistance changed significantly (by one mark for the pigment-printed fabric and by 1.5 marks for the reactive-printed fabric). After this, the mark remained constant until the end of the pilling test. This may have been caused by the different method of printing and the use of a different dyestuff. In pigment printing, the dyestuff distributes only on the surface of the fabric and it does not soak into the fabric inside, i.e., the fabric pattern can be seen only on the right side of the fabric. With reactive printing, the fabric is soaked and the active dyestuff is absorbed into the fabric. The printed pattern slightly saturates into the wrong side of the fabric. The result of the pigment-printed fabric was better because the areas where the pigment dyestuff was on the surface of the fabric were more resistant than those where there was a lack of the pigment dyestuff. The final pilling marks for the fabrics printed using both methods were significantly better than those of the dyed and singed fabrics. No references to the pilling performance of printed fabrics have been found in the existing scientific literature; thus, it can be stated that this research is new and important. The dyestuff did not absorb into the wrong side of the fabric during digital printing; the dyestuff absorption was more superficial, in contrast to the dyed fabrics. Thus, the dyestuff formed a cover on the surface of the fabric and this improved the pilling performance of the fabrics. The pigment-dyed fabrics showed particularly improved pilling resistance because the fabric absorbed the pigment dyestuff less than when the active method was used.

The dynamics of the changes in appearance of all the fabrics with different finishing are presented in Table 2. It can be seen from the pictures in the table that the appearance of all the fabrics already began to change after 125 abrasion cycles. The surfaces of the fabrics fuzzed and pills partially started to form. The appearance of the fabrics worsened gradually after each number of cycles; fuzzing increased on the surface of the fabrics and the number of the pills rose, whereas the fabrics without and with singeing achieved a pilling mark of 2.5 after 2000 abrasion cycles. The appearance of the fabrics printed using both methods changed less. These fabrics showed only moderate pilling of their surfaces (marks 3 or 3.5).

Thus, in summary, it can be stated that the pilling resistance of fabrics and the nature of their changing depends on the finishing used for them.

## 4. Conclusions

Generally, the pilling results of the dyed fabrics were better than those of the grey fabrics. The reason for this may have been that the dyestuff adhered the fuzzes and pills to the surface of the fabric and this led to the better pilling resistance of the dyed fabric. The investigation’s results also showed that even a small amount of synthetic fibers worsens the pilling performance of the fabric.

Singeing influenced the nature of the change in the pilling resistance of the linen/silk fabrics without changing the final pilling resistance mark. These results could have been influenced by the raw material of the fabric, in which two natural fibers (linen and natural silk) of a different nature were blended. Singeing had a greater influence on the fabric with a small amount of synthetic fibers.

The pilling resistance of the printed fabrics was better than that of the grey and dyed fabrics without and with singeing. The reason was that, during dyeing, the entire fabric was immersed in the dye solution and the dyestuff was absorbed into the fabric. During printing, the dye was applied only to the surface of the fabric, improving the pilling resistance of the fabric.

The pilling resistance of pigment-printed fabrics was better than that of the reactive-printed fabrics because of the peculiarities of the type of dyestuff and its penetration into the fabric, i.e., the fabric absorbed the pigment dyestuff less than the reactive one. The reactive dye formed covalent bonds with the fabric so, during dyeing with the pigment dyestuff, it did not create any chemical reactions with the fabric.

The general recommendations will be to use mechanical finishing combined with digital printing for blends of two natural fibers and to use singeing and dyeing for blends of natural and synthetic fibers. These combinations of finishing will be optimal for the given raw materials.

Future studies may include different, new, and unexpected blends of textile fibers, such as linen/wool, linen/alpaca, their blends with other natural and synthetic fibers, etc. They can include such fabrics’ end-use properties, such as pilling and abrasion resistance, and search for means of improving of these properties.

## Figures and Tables

**Table 1:** Evaluation of the pilling marks.

Mark	Description
5	Appearance does not change.
4	Slight fuzzing on the surface and (or) partially formed pills.
3	Medium fuzzing on the surface and (or) medium pilling. Pills of different magnitude and density partially cover the fabric surface.
2	Significant fuzzing and (or) significant pilling. Pills of different magnitude and density cover a large part of the fabric surface.
1	Extremely significant fuzzing on the surface and (or) extremely significant pilling. Pills of different magnitude and density cover the whole fabric surface.

**Table 2:** Appearance of linen/silk fabrics during the pilling test.

Appearance before Test	Appearance after 500 Cycles	Appearance after 2000 Cycles
Grey fabric without singeing		
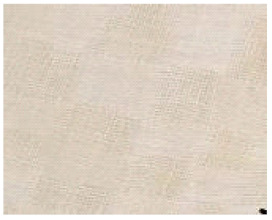	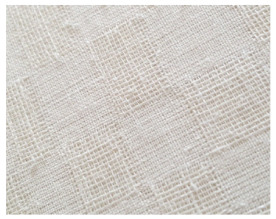	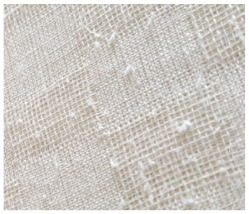
Finished fabric without singeing		
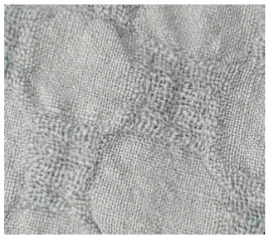	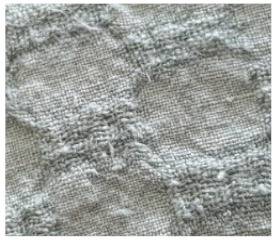	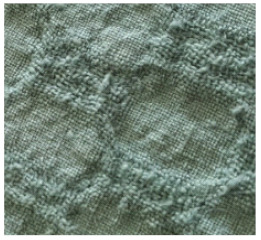
Grey fabric with singeing		
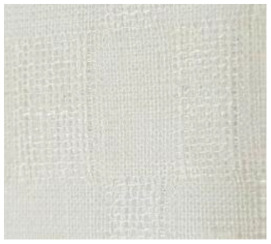	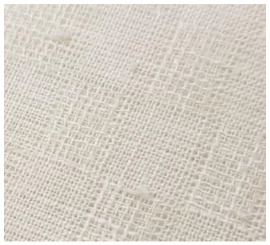	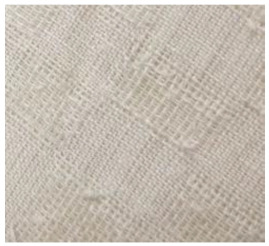
Finished fabric with singeing		
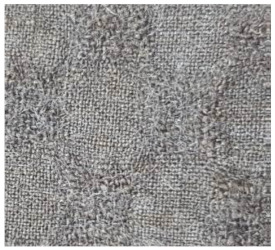	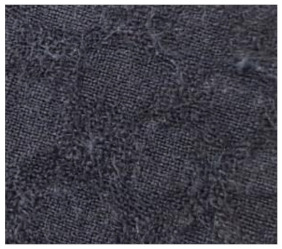	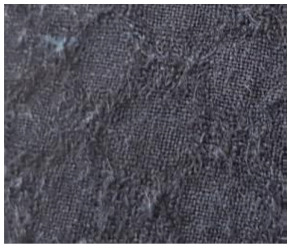
Pigment-printed fabric		
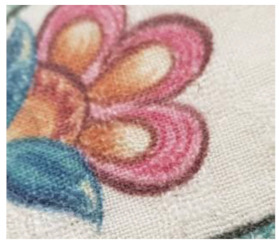	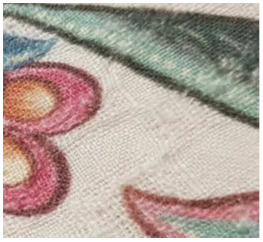	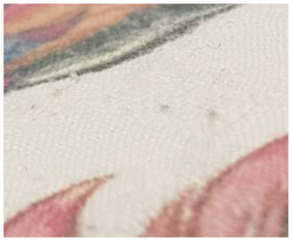
Reactive-printed fabric		
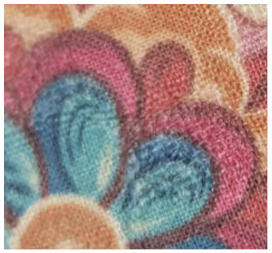	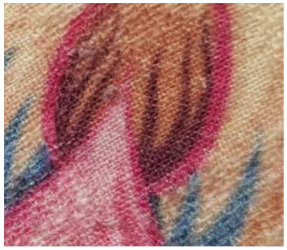	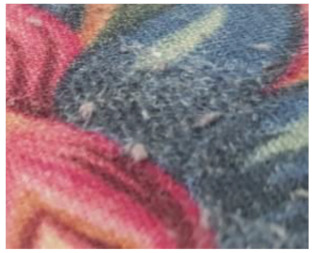

## Data Availability

The data presented in this study are available on request from the corresponding author.
